# Secondary Adrenal Insufficiency as an Immune-Related Adverse Event of Pembrolizumab Therapy

**DOI:** 10.7759/cureus.65163

**Published:** 2024-07-23

**Authors:** Lorena Escaño, Prarthana Desai

**Affiliations:** 1 Internal Medicine, Danbury Hospital, Danbury, USA

**Keywords:** keytruda®, immune-related adverse event (irae), immune check-point inhibitor, pembrolizumab side effect, adrenal insufficiency (ai)

## Abstract

Pembrolizumab is an immune checkpoint inhibitor that blocks the PD1 receptor and is currently used in the treatment of a vast variety of malignancies. Despite adrenal insufficiency already being documented as a rare but potential side effect, its diagnosis is usually delayed due to its vague presentation, increasing the risk of deleterious outcomes.

We present a case of secondary adrenal insufficiency while on pembrolizumab, in which the diagnosis was delayed due to a nonspecific clinical picture. This case highlights the importance of maintaining a high index of suspicion for endocrine dysfunction in patients treated with immune checkpoint inhibitors, as early recognition and appropriate intervention are paramount to preventing serious complications in these patients.

## Introduction

The use of immune checkpoint inhibitors has revolutionized cancer treatment over the past few years. Pembrolizumab, sold under the brand name Keytruda (Merck & Co., Inc., Rahway, NJ, USA), is an FDA-approved immune checkpoint inhibitor directed against the program cell death protein 1 (PD-1) and is currently being used in a vast variety of malignancies such as melanoma, non-small cell lung cancer, and Hodgkin lymphoma [1]. As the use of immune checkpoint inhibitors increases, so do the reports of potential side effects associated with these medications. The activation of the immune system caused by these drugs has been associated with immune-related adverse events (irAEs), which include endocrinopathies such as thyroid disorders and adrenal insufficiency (AI) [2,3].

Pembrolizumab-induced adrenal insufficiency has been documented in the literature; however, its occurrence is still rare, with a reported incidence of less than 1%. Despite its low incidence as an immune-related adverse event, AI can be life-threatening if not diagnosed and treated promptly. However, given its very heterogeneous and vague clinical presentation, a prompt diagnosis can be challenging [4-7].

## Case presentation

We present a 66-year-old male who presented to the emergency department with decreased appetite, nausea, and generalized weakness. His past medical history was remarkable for hypertension, hyperlipidemia, and squamous cell carcinoma of the skin, with recent metastasis to the lungs, for which he was started on pembrolizumab a year prior. 

On arrival, the patient reported nausea, lack of appetite, poor oral intake, and persistent generalized weakness associated with episodes of lightheadedness, mostly when standing. The persistent and worsening symptoms prompted him to present himself to the emergency department for further evaluation. He was seen in the hospital a month prior with similar symptoms, which were thought to be secondary to poor oral intake in the setting of an underlying malignancy. He was managed with intravenous (IV) fluids at the time and home-dose amlodipine was discontinued. 

In the emergency department, the patient was hypertensive, with a blood pressure of 144/68 mmHg, a heart rate of 88 b/m, and a respiratory rate of 15 r/m. Orthostatic vitals were positive. The physical examination was otherwise unremarkable. 

Pertinent laboratory test results were positive for pancytopenia, anion gap metabolic acidosis with ketosis, acute kidney injury, hypernatremia, hyperchloremia, and hypercalcemia, as presented in Table 1. Urinalysis was only remarkable for 3+ ketones, with no findings suggestive of infection. 

**Table 1:** Pertinent laboratory test results on admission

Laboratory test	Laboratory result	Reference value
Glucose	92	70 – 99 mg/dl
Bicarbonate	19	22 – 29 mmol/L
Anion gap	21	10 – 19 mmol/L
Beta-hydroxybutyrate	5.4	0 – 0.4 mmol/L
Lactic acid	1.7	0.5 – 2.2 mmol/L
Creatinine	1.62	0.67 – 1.23 mg/dL
Glomerular filtration rate	47	>60 mL/min
Sodium	147	135 – 145 mmol/L
Potassium	4.4	3.5 – 5.3 mmol/L
Chloride	110	97 – 107 mmol/L
Calcium	11.5	8.6 – 10.4 mg/dl
Hemoglobin	10.3	13.5 – 17 g/dl
Hematocrit	38.8%	38 – 50 %
White blood cells	2,500	3,500 – 10,000
Platelets	138,000	150,000 – 400,000

The patient was admitted to the hospital for further evaluation and management of orthostasis and acute kidney injury thought to be in the setting of starvation ketosis. Initial management included IV fluids and midodrine. However, he continued to have lightheadedness, a poor appetite, and episodes of orthostatic hypotension. Lack of improvement of symptoms led to further workup, which revealed low a.m. cortisol and low adrenocorticotropic hormone (ACTH) consistent with secondary adrenal insufficiency, as seen in Table 2. Given the significantly low a.m. cortisol level (<3 mcg/dl), the cosyntropin stimulation test was deemed unnecessary. The thyroid function panel revealed subclinical hypothyroidism. 

**Table 2:** Laboratory test results indicating secondary adrenal insufficiency and subclinical hypothyroidism ACTH: adrenocorticotropic hormone; TSH: thyroid stimulating hormone

Laboratory test	Laboratory result	Reference value
Morning cortisol	1.1	5 – 25 mcg/dl
ACTH	< 3	7.2 – 63.3 pg/ml
TSH	5.35	0.27 – 4.20 mIntlunits/L
Free T4	0.98	0.80 – 1.70 ng/dl
Free T3	2.7	2.2 – 4 pg/ml
Total T3	126	80 – 200 ng/dl

Endocrinology was consulted. The patient had been receiving Keytruda every six weeks for the past year, which raised suspicions of adrenal insufficiency secondary to pembrolizumab therapy. He was administered solu-medrol 20 mg IV for a total of two doses before being transitioned to a daily oral hydrocortisone regimen. Appetite improved, orthostasis resolved, and midodrine was discontinued. He was discharged home with the plan to finalize pituitary function workup and imaging as an outpatient. 

## Discussion

Pembrolizumab has emerged as a cornerstone in the treatment of various malignancies, offering significant clinical benefits through the enhancement of T-cell-mediated immune responses against tumor cells [1,2]. However, this augmentation of the immune system’s activity can also lead to immune-related adverse events such as thyroid disorders and adrenal insufficiency. Both primary and secondary adrenal insufficiency have been reported as an irAE of pembrolizumab therapy [2,3]. The incidence is relatively low and varies between studies, but it is generally less than 1% and can develop at any time throughout treatment. When this drug is used in combination with other therapies, the incidence of AI may be higher [4-7].

**Figure 1:**
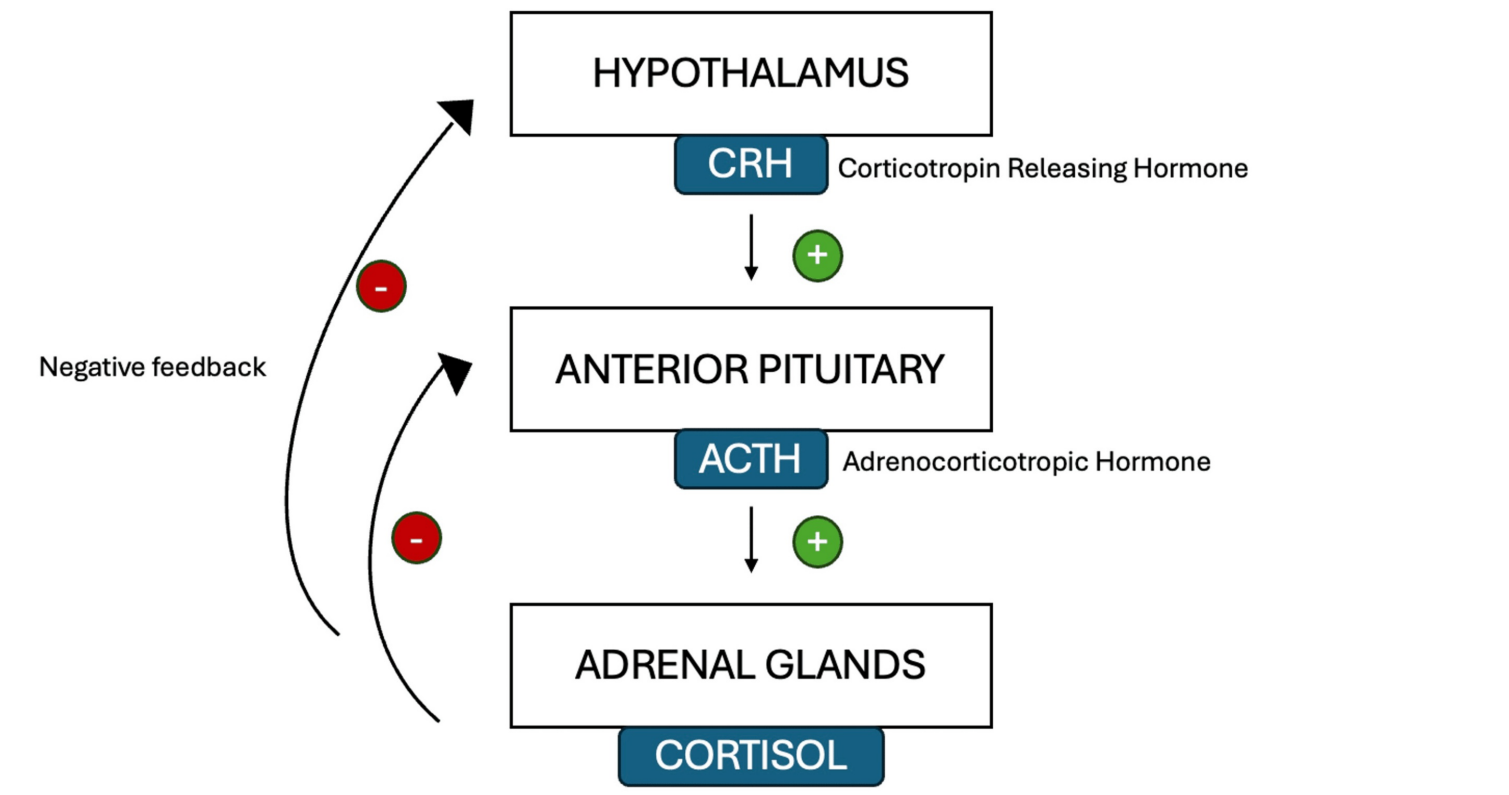
Hypothalamic-pituitary-adrenal axis

The development of adrenal insufficiency in the context of pembrolizumab therapy is attributed to autoimmune-mediated damage to the hypothalamic-pituitary-adrenal axis (Figure 1). Pembrolizumab blocks the interaction between PD-1 and its ligands (PD-L1 and PD-L2), preventing the inhibition of T-cell activity. While this mechanism enhances antitumor immunity, it can also lead to an unregulated immune response against normal endocrine tissues. This autoimmune attack can result in hypophysitis, a condition characterized by inflammation of the pituitary gland, leading to impaired secretion of ACTH and subsequent secondary adrenal insufficiency, as seen in our patient [6-8].

The diagnosis of adrenal insufficiency involves a combination of clinical assessment, laboratory tests, and imaging studies. Low levels of morning cortisol (<5 mcg/dl) are highly suggestive of adrenal insufficiency, and a level <3 mcg/dl confirms the diagnosis, as seen in our patient, whose a.m. cortisol was 1.1 mcg/dl. Elevated ACTH levels indicate primary adrenal insufficiency, while low or normal ACTH levels are concerning for secondary adrenal insufficiency. Confirmatory testing of AI is usually achieved with the cosyntropin stimulation test, which measures the adrenal response to synthetic ACTH. However, the use of this test to discriminate between primary and secondary AI is no longer indicated if morning cortisol and baseline ACTH levels are unequivocal [9]. Our patient had a morning cortisol of 1.1 mcg/dl and an ACTH of <3 pg/ml, making this test unnecessary. Imaging studies such as MRIs of the pituitary can help assess for structural abnormalities and were scheduled to be performed in an outpatient setting [9]. Our patient had no recent use of steroids or any other medication associated with AI and had no recent radiation therapy, making pembrolizumab the likely causative agent of his clinical picture. 

Primary adrenal insufficiency presents with hyponatremia in about 90% of cases and hyperkalemia in about 65% of cases. However, these laboratory abnormalities occur because of aldosterone deficiency, so they are usually absent in patients with secondary adrenal insufficiency, as seen in our patient [9]. The electrolyte abnormalities seen in our patient were most likely secondary to hemoconcentration and were corrected with appropriate fluid resuscitation. 

Our patient presented with symptoms that are frequent in patients with AI, such as generalized weakness, decreased appetite, and nausea. However, these symptoms are nonspecific and can be present in other conditions. He was also hypertensive on arrival, which is unusual, although he did have orthostatic hypotension. Other frequent features, such as hyponatremia and hypoglycemia, were absent in our patient. This vague presentation resulted in delayed recognition of AI as the culprit of his symptoms. 

The nonspecific and insidious presentation of adrenal insufficiency can make the diagnosis challenging [10,11]. In addition to this, routine endocrine screening is not always performed, leading to potential delays in diagnosis, as seen in our patient. Moreover, there is still no formal consensus on screening protocols for patients on immune checkpoint inhibitors, and a multidisciplinary approach is needed to facilitate prompt recognition of these immune-related adverse events [4-10].

## Conclusions

Poor appetite, nausea, generalized weakness, and hypotension are all hallmark features of adrenal insufficiency. However, these symptoms overlap significantly with other conditions, including the common side effects of cancer and its treatment, which resulted in an underestimation of our patient's symptoms during prior admissions. 

This case highlights the importance of always considering adrenal insufficiency as a potential adverse effect of immune checkpoint inhibitors. This will ensure a prompt diagnosis of an endocrine disorder that could lead to deleterious outcomes if left untreated. Further research is needed to better elucidate the exact mechanisms, risk factors, and optimal monitoring protocols for these patients. 

## References

[1] Kwok G, Yau TC, Chiu JW, Tse E, Kwong YL (2016). Pembrolizumab (Keytruda). Hum Vaccin Immunother.

[2] Kumar V, Chaudhary N, Garg M, Floudas CS, Soni P, Chandra AB (2017). Current diagnosis and management of immune related adverse events (irAEs) induced by immune checkpoint inhibitor therapy. Front Pharmacol.

[3] Wang PF, Chen Y, Song SY (2017). Immune-related adverse events associated with anti-PD-1/PD-L1 treatment for malignancies: a meta-analysis. Front Pharmacol.

[4] Ida H, Goto Y, Sato J (2020). Clinical characteristics of adrenal insufficiency as an immune-related adverse event in non-small-cell lung cancer. Med Oncol.

[5] Ariyasu R, Horiike A, Yoshizawa T (2017). Adrenal insufficiency related to anti-programmed death-1 therapy. Anticancer Res.

[6] Zilberman S, Rafii DC, Giunta J (2023). Pembrolizumab-induced adrenal insufficiency presenting eight months after cessation of treatment. Cureus.

[7] Barroso-Sousa R, Barry WT, Garrido-Castro AC, Hodi FS, Min L, Krop IE, Tolaney SM (2018). Incidence of endocrine dysfunction following the use of different immune checkpoint inhibitor regimens: a systematic review and meta-analysis. JAMA Oncol.

[8] Paepegaey AC, Lheure C, Ratour C (2017). Polyendocrinopathy resulting from pembrolizumab in a patient with a malignant melanoma. J Endocr Soc.

[9] Melmed S, Auchus RJ, Goldfine AB (2020). The Adrenal Cortex - Chapter 13. Williams Textbook of Endocrinology 15th edition.

[10] Sonehara K, Tateishi K, Araki T, Komatsu M, Akahane J, Yamamoto H, Hanaoka M (2021). Pembrolizumab-induced adrenal insufficiency in patients with untreated advanced non-small cell lung cancer: a case series. Case Rep Oncol.

[11] Shaikh S, Nagendra L, Shaikh S, Pappachan JM (2023). Adrenal failure: an evidence-based diagnostic approach. Diagnostics (Basel).

